# Mucinous Cystic Neoplasm of the Cystic Duct: A Rare Location of a Rare Entity

**DOI:** 10.7759/cureus.14377

**Published:** 2021-04-09

**Authors:** Nicholas J Caldwell, Ilham Farhat, Sarag Boukhar

**Affiliations:** 1 Pathology, Carver College of Medicine, University of Iowa, Iowa City, USA; 2 Pathology, University of Iowa Hospitals and Clinics, Iowa City, USA

**Keywords:** mucinous cystic neoplasm, cystic duct, biliary cystadenoma

## Abstract

Mucinous cystic neoplasms (MCNs) are uncommon cystic lesions that arise in the liver and biliary system (MCN-LBS) and the pancreas (MCN-P) and rarely arise from the extrahepatic biliary system. Histologically, these lesions are defined by the presence of variably mucin-producing epithelium with ovarian-like, hypercellular mesenchymal stroma. Herein, we present a case of extrahepatic MCN-LBS in a 51-year-old woman. This lesion arose from the cystic duct and was removed via laparoscopic cholecystectomy. Histologic examination confirmed the diagnosis. To our knowledge, this is the third case report of an MCN-LBS arising from the cystic duct in the English literature. In this article, we review clinical and histologic characteristics of MCNs and present two other reports of MCN-LBS of the cystic duct.

## Introduction

Mucinous cystic neoplasms (MCNs) of the liver and biliary system (MCN-LBS) are rare, benign cystic lesions with a reported incidence of 1 case per 20,000 to 100,000 person-years [1]. Per World Health Organization (WHO) diagnostic criteria, MCNs occur exclusively in females. Patients typically present in the fifth to sixth decades of life with symptoms related to the anatomic location and space-occupying nature of the lesion [2, 3]. Macroscopically, these are multilocular cystic lesions without communication to the biliary duct system. Microscopically, the cystic spaces are typically lined by cuboidal to columnar, variably mucin-producing epithelium (unless surface-denuded) overlying a hypercellular bland spindle cell proliferation resembling ovarian stroma, known as ovarian-like or ovarian-type stroma. MCN-LBS occur predominantly within the liver and intrahepatic biliary system, and there is a variably reported, yet low, percentage of these lesions occurring in the extrahepatic biliary system [4, 5]. Herein, we present a case of extrahepatic MCN-LBS arising from the cystic duct.

## Case presentation

**Figure 1:**
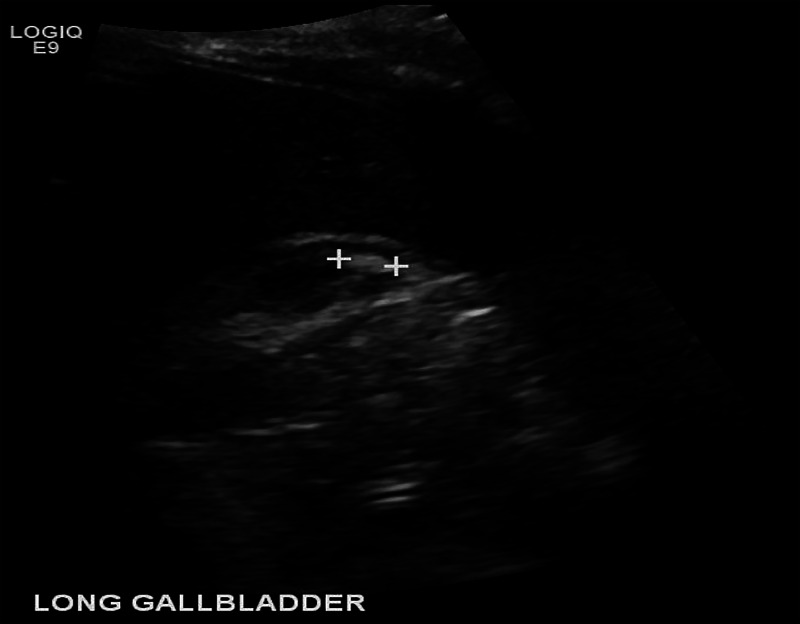
Abdominal ultrasound Preoperative ultrasonography highlighting the assumed non-mobile gallstone (between the plus signs) within the gallbladder fundus.

The patient is a 51-year-old woman who is a former smoker with a past medical history of hypertension and gastrointestinal reflux disease. She initially presented with a few months of right upper quadrant abdominal pain with subsequent ultrasonography reporting a 1 cm non-mobile stone within the fundal region of the gallbladder (Figure 1).

**Figure 2:**
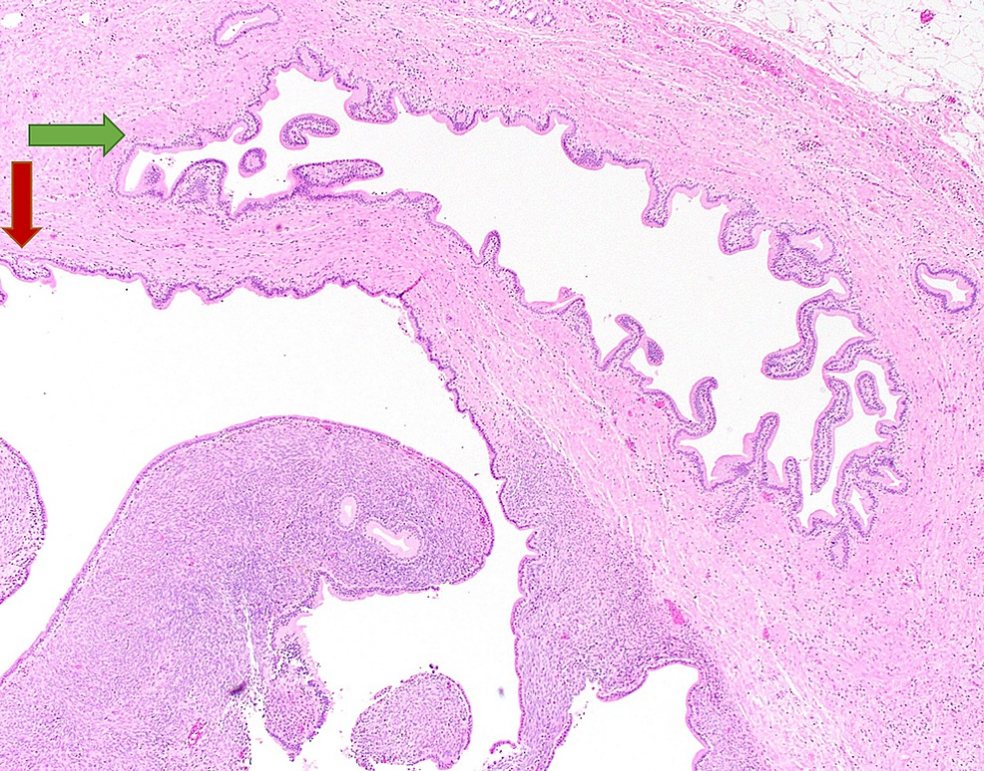
Mucinous cystic neoplasm arising from the cystic duct A low-power view demonstrating a cystic lesion on the lower left (red arrow), lined by bland mucinous epithelium with underlying hypercellular stroma, and cystic duct lumen on the upper right (green arrow). Note the absence of luminal communication between the cystic duct and the adjacent cystic lesion (H&E, x4 magnification). H&E = hematoxylin and eosin

**Figure 3:**
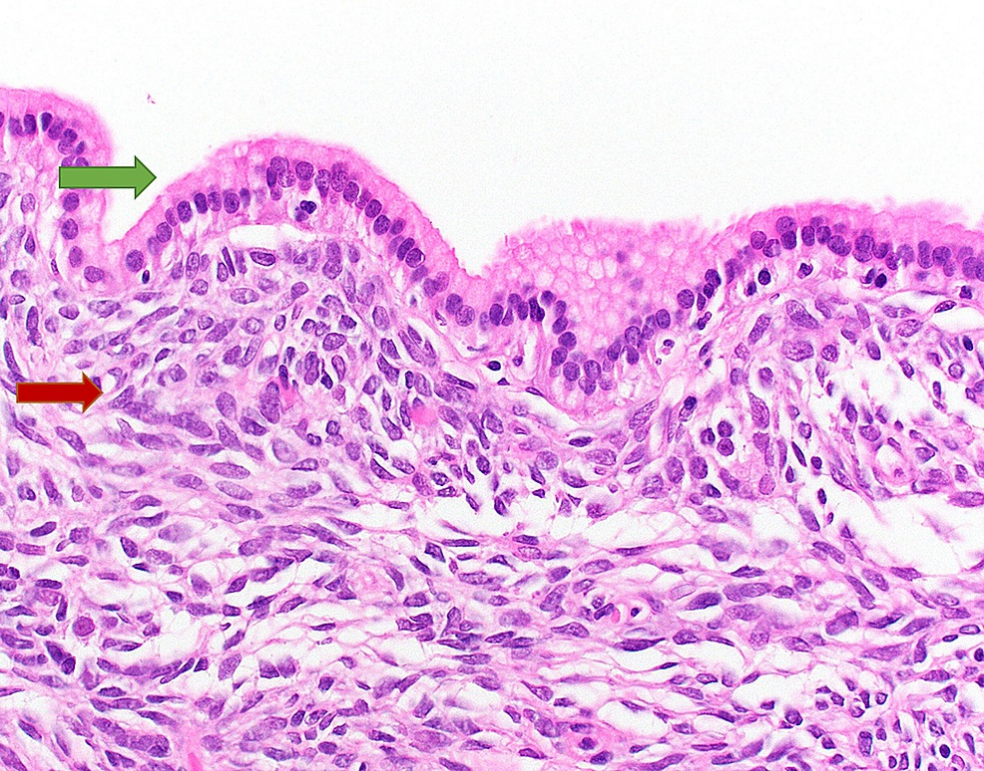
Mucinous cystic neoplasm arising from the cystic duct A high-power view demonstrating the single layer of mucinous cuboidal to columnar epithelium (green arrow) and the pathognomic ovarian-like, mesenchymal stroma (red arrow) formed of densely cellular bland spindle cells (H&E, x40 magnification). H&E = hematoxylin and eosin

She eventually received a laparoscopic cholecystectomy revealing a grossly unremarkable gallbladder without gallstones. Microscopic evaluation revealed a small cystic lesion within a cystic duct section involving the surgical margin. The cyst wall showed a bland, cuboidal to columnar, mucinous epithelial lining overlying an ovarian-like, mesenchymal stroma (Figures 2, 3).

**Figure 4:**
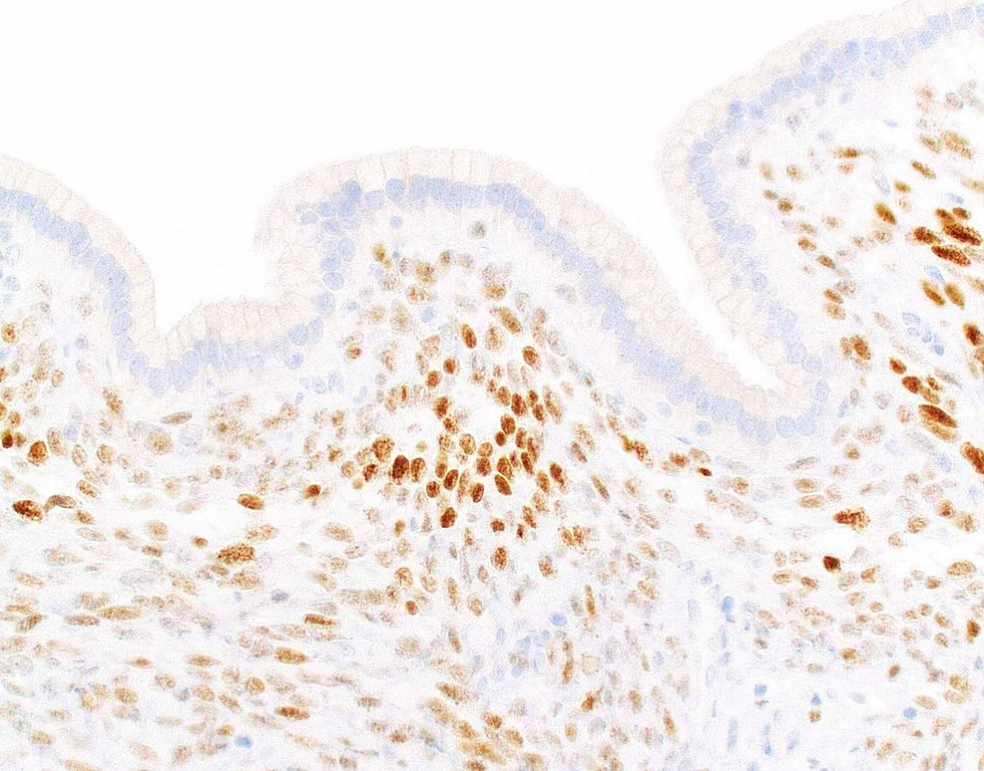
Mucinous cystic neoplasm arising from the cystic duct (IHC) A high-power view of the lesion from the same area depicted in Figure 2 shows nuclear positivity (brown color staining) of the stromal cells (ovarian-like stroma) to ER immunostaining (DAB, x40 magnification). IHC = immunohistochemistry, ER = estrogen receptor, DAB = 3-3’-diaminobenzidine

The immunoreactivity of the ovarian-like stroma to estrogen receptor further confirmed the morphologic impression of mucinous cystic neoplasm (Figure 4).

**Figure 5:**
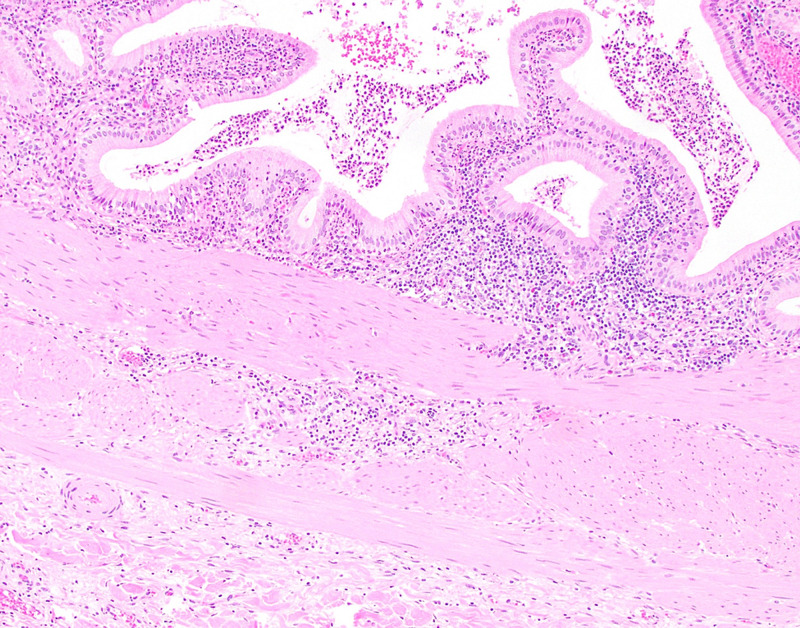
Chronic active cholecystitis A medium-power view of the gallbladder demonstrating mixed mucosal inflammation extending to the underlying, mildly thickened, muscular layer (H&E, x10 magnification). H&E = hematoxylin and eosin

The entire specimen was subsequently submitted for examination and sections of the background gallbladder revealed chronic active cholecystitis with no evidence of tumor involvement (Figure 5). No high-grade dysplasia or associated carcinoma was identified. She is currently under medical observation with periodic imaging. Surveillance magnetic resonance imaging (MRI) obtained 4 and 16 months postoperatively were negative for disease recurrence, and as of her last follow-up (16 months postoperatively), she was asymptomatic.

## Discussion

MCNs most commonly arise in the liver and pancreas and rarely arise from the extrahepatic biliary system [2, 6]. As evident grossly and radiographically, these lesions are multiloculated with variable presence of loculations, septae, calcifications, papillary projections, and small mural nodules [7, 8]. The reported average lesion size is approximately 11 cm (range 1.4-30 cm) [2, 3]. MCNs rarely have associated focal high-grade dysplasia and/or invasive carcinomas that are unable to be detected preoperatively [2, 3, 6, 9]. Studies reporting cases of MCN-LBS with associated dysplasia are presented in Table 1. Extensive sampling of these lesions is recommended and it is the authors’ practice to submit the entire lesion for microscopic examination whenever possible. Because of the risk of dysplasia and carcinoma, management typically involves complete surgical excision with an excellent prognosis with complete lesion removal [10].

**Table 1:** Reported incidences of dysplasia and carcinoma in mucinous cystic neoplasm of liver and biliary system MCN-LBS = mucinous cystic neoplasms of the liver and biliary system

Author (year)	Findings of Dysplasia in MCN-LBS
Quigley (2018) [2]	2 (6%) of 36 MCN-LBS with high-grade dysplasia with microscopic foci of invasion
Zhelnin (2017) [3]	3 (9%) of 32 MCN-LBS with invasive carcinoma, 1 (3%) of 32 MCN-LBS with focal high-grade dysplasia
Fujikura (2017) [9]	1 (13%) of 8 MCN-LBS with high-grade dysplasia
Zen (2011) [6]	2 (7%) of 29 MCN-LBS with borderline malignancy, 1 (3%) of 29 MCN-LBS with carcinoma in situ

Essential histologic criteria for MCN-LBS - initially introduced in 2010 and recently restated in 2019 - is listed in the World Health Organization (WHO) Classification of Tumours [1, 11]. Chief among these is the requirement for demonstrable ovarian-like, mesenchymal stroma with at least focal positively for immunohistochemical stains estrogen receptor (ER) and progesterone receptor (PR). Like the presence of an associated carcinoma, this stroma may be focal, thus requiring adequate sampling of the lesion to confirm or refute its presence [2]. Additional criteria include non-communication of the lesion with the bile duct system and neoplastic epithelium with at least partial mucin staining. Interestingly, the WHO also lists female exclusivity of the lesion as an essential diagnostic feature of the entity. Arguments against this diagnostic criteria take aim at the need for mucinous epithelium and lack of bile duct communication; authors have proposed that lesions without these characteristics but with the presence of ovarian-like, mesenchymal stroma could potentially be considered as MCNs [12, 13, 14]. Additionally, there is literature describing MCNs in men [3].

Reviewing scientific literature regarding this entity is challenging due to inconsistent lesion nomenclature and incomplete mention of the current essential histologic diagnostic criteria for MCNs. Prior to 2010, the entity of MCN-LBS did not exist; rather, cystic lesions of the liver and extrahepatic biliary system were commonly referred to as “biliary cystadenomas.” Lesions referred to as biliary cystadenomas were typically cystic lesions with varying epithelium with or without the presence of the ovarian-like stroma that is so crucial for the diagnosis of MCN today. As such, the findings of larger sample studies of biliary cystadenoma cannot easily be applied to MCN-LBS [5, 15, 16].

Reports of biliary cystadenomas and MCNs do not universally describe the histopathologic criteria needed to make a current diagnosis of MCN. This is best demonstrated with a comparison of two literature reviews regarding MCNs of the gallbladder; while both manuscripts were published in 2018, one (Sugawara et al.) reports three cases of gallbladder MCNs [17] while the other (Rivero-Soto et al.) reports sixteen [4]. However, many of the cases cited by Rivero-Soto’s manuscript either report a lack of ovarian-like stroma or are missing evidence needed for current MCN diagnosis. Our review of the literature revealed at least four gallbladder MCNs - the three described in Sugawara et al.’s manuscript and the one reported in Rivero-Soto et al.’s manuscript.

As alluded to previously, there is scant literature describing MCNs arising in the extrahepatic biliary system that fulfill the essential histopathologist diagnostic criteria. Among these reports, there are only two case reports of MCNs arising from the cystic duct [17, 18]. Clinical and pathologic data from these two reports alongside our presented case are displayed in Table 2. All cases occurred in women and had histologically proven ovarian-like stroma. Mucinous epithelium was noted in two of the cases. Two of the lesions’ sizes were on the lower end of the reported range for MCN size; compared to an average MCN-P (pancreas) size of 6.6 cm and MCN-L (liver) size of 11.2 cm, these lesions were 1.8 cm and 1.0 cm in their greatest dimension [3]. These small lesions likely became symptomatic due to their proximity to the cystic duct. The report of an 8.5 cm MCN of the cystic duct had an accompanying gallbladder component, which likely explains its increased size and the patient's lack of symptoms. None of the three cases had any associated high-grade dysplasia or invasiveness, and all were treated via surgical removal without any noted recurrence at follow-up.

**Table 2:** Reported Cases of Mucinous Cystic Neoplasm arising from the Cystic Duct RUQ = right upper quadrant, IHC = immunohistochemistry, OLS = ovarian-like stroma, ER = estrogen receptor, PR = progesterone receptor

Author, Year	Marcotte, 2013 [18]	Sugawara, 2018 [17]	Current Case, 2021
Patient Demographics	50-year-old woman	70-year-old woman	51-year-old woman
Presenting symptoms	Nausea, vomiting, postprandial RUQ abdominal pain	Asymptomatic	RUQ abdominal pain
Anatomic location	Cystic Duct	Gallbladder and Cystic Duct	Cystic Duct
Size	1.8 x 1.5 x 1.4 cm	8.5 x 6.0 cm	1.0 cm
Gross Appearance	Multiloculated, cystic	Multiloculated, cystic	Normal
Epithelium	Not described	Cuboidal to columnar mucin-producing cell	Columnar mucinous cell
Epithelial Dysplasia?	Not described	Low-grade dysplasia present	Absent
Stroma	OLS	OLS	OLS
IHC of OLS	Not described	ER negative, PR positive	ER positive
Communication with biliary structures	Absent	Absent	Absent
Management	Laparoscopic Cholecystectomy	Cholecystectomy	Laparoscopic Cholecystectomy
Long-term Outcome	Not described	No recurrence eight years postoperatively	No recurrence 14 months postoperatively

## Conclusions

MCNs are a distinct clinical entity distinguished from other cystic lesions by their ovarian-like, hypercellular mesenchymal stroma, among other criteria. While most of these lesions arise from the liver and biliary system (MCN-LBS) and pancreas (MCN-P), these lesions occasionally arise in the extrahepatic biliary system. We reported the third case of MCN-LBS arising from the cystic duct and discussed the clinical and histopathologic characteristics of the lesion. MCN-LBS arising from the cystic duct are histologically identical to those of the liver; however, it may present at a smaller size due to their proximity with critical biliary structures. Continued acknowledgement of WHO diagnostic criteria and nomenclature for this entity is important to further define clinical characteristics of this lesion. For this reason, the authors support the WHO’s recommendation for discontinuation of the historic term “biliary cystadenoma.”
